# “If I Don’t Eat Enough, I Won’t Be Healthy”. Women’s Experiences with Gestational Diabetes Mellitus Treatment in Rural and Urban South India

**DOI:** 10.3390/ijerph17093062

**Published:** 2020-04-28

**Authors:** Karoline Kragelund Nielsen, Thilde Vildekilde, Anil Kapur, Peter Damm, Veerasamy Seshiah, Ib C. Bygbjerg

**Affiliations:** 1 Diabetes Prevention, Health Promotion Research, Steno Diabetes Center Copenhagen, Niels Steensens Vej 6, 2820 Gentofte, Denmark; 2 Global Health Section, Department of Public Health, University of Copenhagen, Oester Farimagsgade 5, 1014 Copenhagen, Denmark; 3 World Diabetes Foundation, Krogshoejvej 30A, 2820 Bagsvaerd, Denmark; 4 Center for Pregnant Women with Diabetes, Department of Obstetrics, Rigshospitalet, Blegdamsvej 9, 2100 Copenhagen, Denmark; 5 Department of Clinical Medicine, University of Copenhagen, Blegdamsvej 3B, 2200 Copenhagen, Denmark; 6 Dr. Seshiah Diabetes Research Institute and Dr. Balaji Diabetes Care Centre, 729 Poonamallee High Road, Aminjikarai, Chennai, Tamil Nadu 600029, India

**Keywords:** gestational diabetes mellitus, India, treatment, access to health care, qualitative research

## Abstract

Gestational diabetes mellitus (GDM) is associated with a range of adverse pregnancy outcomes as well as increased risk of future type 2 diabetes and cardiovascular disease. In India, 10%–35% of pregnant women develop GDM. In this study, we investigated women’s experiences with the dietary and pharmaceutical treatment for GDM in rural and urban Tamil Nadu, India. Semi-structured interviews were conducted with 19 women diagnosed with GDM. Data were analyzed using qualitative content analysis. Three overall aspects were discovered with several sub-aspects characterizing women’s experiences: emotional challenges (fear and apprehension for the baby’ health and struggling to accept a treatment seen as counterintuitive to being safe and healthy), interpersonal challenges (managing treatment in the near social relations and social support, and coordinating treatment with work and social life), and health system-related challenges (availability and cost of treatment, interaction with health care providers). Some aspects acted as barriers. However, social support and positive, high-quality interactions with health care providers could mitigate some of these barriers and facilitate the treatment process. Greater efforts at awareness creation in the social environment and systemic adjustments in care delivery targeting the individual, family, community and health system levels are needed in order to ensure that women with GDM have the opportunity to access treatment and are enabled and motivated to follow it as well.

## 1. Introduction

Gestational diabetes mellitus (GDM) increases the risk of a range of adverse pregnancy outcomes such as macrosomia, caesarean section, obstructed labor, and preeclampsia, [1,2] which are among the leading causes of maternal mortality [3]. It also increases the risk of future type 2 diabetes and cardiovascular disease in both the mother and her offspring [4,5,6,7]. Evidence suggests that timely detection and treatment (diet, sometimes in combination with insulin or metformin) can reduce the risk of these adverse outcomes and thus improve maternal and offspring health [8,9,10,11].

According to estimates from the International Diabetes Federation, more than 20 million live births are affected by hyperglycemia in pregnancy annually; 84% these are GDM cases [12]. The majority of these cases occur in low- and middle-income countries. However, limited evidence is available to inform policy makers, clinicians and women at reproductive age how to best address the challenge of GDM in low- and middle-income countries [13,14]. In India, 10%–35% of pregnant women develop GDM [15,16], and the condition has therefore received increasing attention from health policy makers, researchers and clinicians in the last decade. In the South Indian state Tamil Nadu, universal screening and testing for GDM, using the Diabetes In Pregnancy Study Group India criteria, was introduced as part of routine antenatal care in 2007 [17]. Testing is usually performed in 24th–28th weeks of gestation but may also be performed earlier or later in pregnancy. Treatment for GDM consists of dietary management, supplemented with pharmaceutical intervention (insulin or metformin) if glycemic control is not obtained.

In a previous study, we documented factors influencing timely initiation and completion of the GDM screening and diagnostic process in Tamil Nadu [18]. One key finding from the study is the importance of the family and the prominence of motherhood and its social implications within the family as a motivator for testing. Notions of motherhood, childbearing and pregnancy in India have been described in a number of ethnographic works, documenting the pressures placed on women to give birth to a healthy baby and thus continue the family’s lineage [19,20,21]. Consequently, numerous rites are followed to avoid any harm to the fetus or newborn—some of which include the avoidance of certain types of food. Many of these are related to the concepts of ‘hot’ and ‘cold’ foods, which are quite widespread in India. The underlying criteria for classifying foods as either hot or cold remain unclear and vary considerably from area to area but are generally understood as depending on the effects the food will have on the person eating it [22]. However, traditional beliefs or lay perceptions regarding harmful and beneficial practices, including dietary practices, for women during pregnancy do not always conform with biomedical notions and recommendations [22]. Since GDM is a condition that can have harmful effects on both the mother and offspring, and which has a treatment requiring adherence to a fairly strict regimen, it is important to understand how the required treatment practices are perceived and experienced by the women. Therefore, in this study, we investigated women’s experiences with the dietary and pharmaceutical treatment for GDM in rural and urban Tamil Nadu, India.

## 2. Materials and Methods

### 2.1. Study Area

This was a qualitative study conducted in two settings in Tamil Nadu, India: the rural district of Thiruvallur and the state capital Chennai. Thiruvallur district is located approximately 40 km north of Chennai city. Depending on traffic, it takes 2–3 h to travel from Thiruvallur to Chennai by public bus. While Chennai is characterized by a growing middle class and economic growth, especially in the IT and manufacturing sectors, inhabitants in Thiruvallur district mainly work as farmers, daily wagers or workers at the local factories—although some also work in Chennai. The villages in Thiruvallur district are connected to the main roads via small paved or dirt roads.

### 2.2. Interviews with Women Diagnosed with GDM

Data were collected between September 2013 and March 2014 as part of a larger study on GDM [23]. In the two settings, we conducted in-depth interviews with a total of 19 women diagnosed with GDM: 10 from Thiruvallur and nine from Chennai (see Table 1 for study participant characteristics). Sampling was continued until data saturation was reached, i.e., where no new stories and categories emerged from the interviews. In Thiruvallur, the women were identified via a public primary health center, and in Chennai they were identified via a private diabetes hospital. Recruitment was conducted by a trained female Tamil speaking research assistant, who was not affiliated with the health system. She approached the women in person or via telephone, informed them about the study and invited them to participate in an interview. If they agreed, an appointment for an interview was made or the interview was carried out immediately, depending on the woman’s preferences. Prior to the interview, permission to audio record the interviews and verbal informed consent were obtained from all the participating women. The data for this article stem from interviews with both women who were pregnant and women who had already given birth. In all interviews, women were asked questions about experiences of treatment during pregnancy. Post-pregnancy interviews were conducted as part of a larger study in which experiences of postpartum testing were also investigated (not covered in this article). Seven women were interviewed both during their pregnancy and again after their delivery. KKN carried out all interviews; most interviews were carried out with the help of a research assistant who did English–Tamil translation. Two of the women spoke English fluently and preferred to do the interview directly in English.

The age of the interviewed women ranged from 24 to 38 years. All the women from Thiruvallur identified themselves as housewives and only one had an educational level beyond the 10th grade. Among the women from Chennai, five identified as housewives and four had formal employment. Seven of the women in Chennai had graduate or postgraduate degrees. All women were on dietary treatment for their GDM during pregnancy. Two of the women in Thiruvallur had also been instructed to take insulin and one was given insulin when admitted at the hospital for the delivery. In contrast, all nine women from Chennai were also on some form of pharmaceutical treatment, either insulin or metformin, in addition to the dietary treatment.

A semi-structured interview guide was used in the interviews. The guide contained mainly open-ended questions focusing on the women’s perceptions of GDM, the treatment and pregnancy in general, as well as the role of their family and health care providers, and their experiences with following the treatment.

### 2.3. Data Analysis and Ethics

The data analysis followed an inductive-iterative approach and the process of qualitative content analysis [24]. All interviews were transcribed verbatim and translated into English by a research assistant fluent in Tamil and English. Samples of the translations were checked by another local researcher, and the research assistant and KKN thoroughly discussed all English version transcripts to clarify any unclear translations. The transcripts were read through and coded by KKN. Codes were then reviewed, compared and organized into categories. The coding process was carried out using the software program Nvivo 10 (QSR International Pty. Ltd. 1999–2012, Melbourne, Australia). The coding focused on displaying manifest content describing the women’s concrete experiences with following or obtaining treatment. Thus, closeness to the text was sought, i.e., focusing on concrete descriptions and interpretations [25]. The data were organized and presented according to three main categories and six sub-categories, each acting as either barriers or facilitators to the treatment.

Ethical approval for the study was given by the Institutional Ethics Committee at Dr. V. Seshiah Diabetes Research Institute and Dr. Balaji Diabetes Care Centre in Chennai.

## 3. Results

Based on the women’s experiences, three overall categories of treatment challenges were identified: (1) emotional challenges at the individual level, including the two sub-categories of fear and apprehension for the baby’s health; and treatment experienced as a struggle and as counterintuitive to what is considered safe and healthy; (2) interpersonal challenges experienced in the near social domain, including two sub-categories of managing treatment in near social relations and social support; and coordinating treatment with work and social life; and (3) health system level challenges, including two sub-categories of experiences with availability and cost; and experiences with interaction with health care providers.

### 3.1. Emotional Challenges at the Individual Level

#### 3.1.1. Fear and Apprehension for the Baby’s Health

Most of the women described being surprised when diagnosed with GDM, and that they had considerable unpleasant emotional reactions, including shock, fear and sadness because they worried about the consequences of the condition on the baby’s health. Ten of the women reported previous experiences of abortions, stillbirths, loss of a child or having had difficulties conceiving, which had caused and added to their feelings of great sadness and anxiety. Avoiding any potential harm to the new baby was verbalized as the core motivating factor for following the treatment by most women—they had to do it ‘for the sake of the baby’.

‘*I followed my diet only for the sake of the baby. There is nothing more than my baby’s health, so I gave up everything for my baby.*’

(Woman no. 14, Chennai, had two children and had experienced one abortion)

The women described the situation with gravity and obvious emotional strain, which stemmed from receiving information from the health care providers about a range of adverse pregnancy outcomes that could result from a mismanaged GDM. One 26-year-old woman from Chennai explained:

‘*Initially I was so scared, I was in tears. […] That was not something I expected. […] The first thing that went to my mind was ‘what about the baby? What happens?’ [...] [The doctor] said all the extreme things [that could happen] and that really worried me. She said that if the sugar levels are not controlled properly the baby’s organs—the legs, foot, hands and all—may not develop properly. She gave me all the extremes. So that worried us a lot till we had the first scan. […]. So, I mean, whenever I get tempted to eat a little bit, then I will just think about the baby and that I don’t want anything to happen.*’

(Woman no. 6, Chennai)

Thus, while the potential risks of a GDM pregnancy to the baby’s health generally motivated the women to follow the diet, it was accompanied with substantial emotional impact; worries, shock, fear, and concerns followed the women during their pregnancies.

#### 3.1.2. Treatment Experienced as a Struggle and as Counterintuitive to What Is Considered Safe and Healthy

All the women noted that they experienced the treatment as a struggle. Various accounts of hardship or discomfort in following the treatment were described by the interviewed women. In connection to diet, cravings for certain ‘forbidden’ food items (according to health care providers’ advice) were especially highlighted. Reduction in rice intake is one of the main components of the dietary recommendations given to women with GDM in India, and all the women reported being aware of this recommendation. However, rice is consumed as the main staple food in large parts of India, and Tamil Nadu is no exception. Thus, prior to the diagnosis, the women would typically eat three meals a day, with all meals including rice. After the diagnosis, they had been instructed by health care providers to only eat rice once a day and to split their food into several smaller meals in order to regulate their blood glucose levels. However, rice was described by the women as giving them stamina and strength and was perceived as necessary to nourish the baby and to carry out the women’s daily household work. Not being allowed to eat rice was therefore met with skepticism and concern by several of the women. Many women complained that the recommended diet made them hungry, tired, and weak. This created an additional emotional burden and worry about the baby’s health.

‘*I felt it difficult to control because I was worried that my weight was reducing and something may happen to the baby. If I don’t eat enough, I won’t be healthy… I am feeling that the weight of the baby also would decrease because my weight is decreasing. If the baby is thin the baby will have less immunity power and it will have a lot of health problems.*’

(Woman no. 18, Thiruvallur)

Thus, the dietary recommendations given out by health care providers were at times counterintuitive to local and individual perceptions of appropriate foods considered good and healthy during pregnancy. Many of the situations related to diet specifically arose due to local lay health perceptions about food benefits and avoidance during pregnancy, including the traditional hot/cold dualism practiced in Tamil Nadu. The women reported being advised by the health care providers to avoid sugar, fruit juices and certain fruits due to their sugar contents. However, for some women, some of these foods were associated with benefits to the baby according to local lay diet advice. Woman 14 from Chennai noted:

‘*I had to avoid drinking juices. In the first delivery I drank a lot of juices, but for this delivery I controlled it a lot. But I felt that since I drank all those juices in the first pregnancy my baby was fair and healthy, so I felt it difficult controlling drinking juices.*’

(Woman no. 14, Chennai)

Other food items, which were recommended in the official diet plans, were, on the other hand, not considered safe and healthy to eat by the women according to their local lay health perceptions. One woman (Woman no. 5) from rural Thiruvallur, for instance, described that the doctor had told her to include a lot of vegetables and greens with high water content in her diet, but that she would not eat them as she believed the vegetables to be classified as ‘cold foods’ and therefore would risk giving her a cold.

However, urban women in particular also noted that it had become easier to follow the dietary treatment as they got ‘used to it’. A few of the women even described that the new diet eventually was a positive turn in their life, and they appreciated the positive effect it had:

‘*Following the diet, I am fine with it, because I feel really light and better now. I could have been like 10 kilograms more overweight by now had it not been for GDM. So, I feel it is a blessing in disguise for me. Really—because I have been eating healthy food, and a healthy diet.*’

(Woman no. 6, Chennai)

Considerable emotional concerns were also expressed regarding the pharmaceutical treatment of GDM. All ten women, urban as well as rural, taking insulin said they felt uneasy and apprehensive about the injections and found them to be painful.

Furthermore, concerns about the side-effects of the medication, whether insulin or metformin, were noted among a couple of women. These women cited a general notion and instructions from health care providers that no medication should be taken during pregnancy due to the risk of side-effects.

‘*It took me nearly ten days to start it [metformin] because I was thinking is it really necessary? Because I was worried, it is category B [categorization to indicate the potential of a drug to cause birth defects if used during pregnancy], so I was worried about that. So, I spoke to my obstetrician and a few of my other friends who were also diagnosed and who had also taken it, and then I was convinced.*’

(Woman no. 10, Chennai)

Thus, concerns about side-effects caused concerns about taking the medication, which at times resulted in reduced or delayed adherence to treatment. Woman no. 10 had a postgraduate degree and appeared to have a high health literacy level, which probably assisted her in seeking information and eventually feeling comfortable enough to initiate the treatment. However, the concerns about side-effects from medications were also present among women with less educational attainment. Among these women, the concerns seemed more persistent, although all reported taking the medications, at least to some extent.

### 3.2. Interpersonal Challenges Experienced in the Near Social Domain

#### 3.2.1. Experiences with Social Relations and Support

Underlying and influencing most of the women’s experiences with the treatment is the aspect of social relations and support. The women’s family, friends, colleagues, and neighbors played either a mitigating or aggravating role in the various challenges in GDM treatment. The family and other social relations influenced the women’s perception of the treatment as ‘good’ or ‘bad’ for the baby, and they influenced the women’s ability to follow the treatment. Woman no. 10, who talked about the challenges she faced in following the diet when she was at work, also noted that:

‘*My colleagues are very helpful, so I found that even the days where I am not able to carry extra snacks with me, they usually help me out. They bring biscuits or we take the time out to take the break. They are very helpful.*’

(Woman no. 10, Chennai)

The support of the family in following the diet was also a widely noted aspect, both in terms of providing practical support, e.g., purchasing and preparing the food, as well as supporting the women to adhere to the advised diet. This not only limited the practical work associated with the treatment, as only one type of meal had to be cooked instead of two, but also limited the women’s exposure to temptation of taking foods/drinks that were not recommended. Women with scant support from their family noted that it ‘might have been easier’ if they had more help. Woman no. 3 from Thiruvallur had a marriage that was not approved by the families, who therefore had disowned the couple.

‘*Since we are alone, I have to do everything on my own. So, I am doing all the household work. Maybe if I was staying with someone then there might be things that I won’t have to do. They would have given me rest and told me not to do any household work, they would have told me to take care of my health. But since I don’t have anyone with me, I do all the household work.*’

(Woman no. 3, Thiruvallur)

In terms of availability and cost, the investment that was required, as also described later, meant it was paramount that the women’s treatment was considered a priority by the family. Even without financial difficulties, access was sometimes controlled by the family dictate and therefore not freely available to the women. Woman no. 19 described the difference in terms of availability depending on whether she was staying in her in-law’s house or her parents’ house:

‘*In my in-laws’ house I was not able to ask freely what I wanted because they had to make it. In my mom’s place I can ask her anything and she can make it at any point in time. […] I require a kind of food that only I am taking, but since everyone else would like to have some other kind of food they [her in-laws] also force me to take that food on that day. Instead of making special food for me, they made me what was cooked for the rest of the family.*’

(Woman no. 19, Chennai)

For the ten women living with their in-laws, the husband would sometimes play a mitigating role in such circumstances and request his mother to provide certain foods for the woman. The husbands were likewise described by the women as being important in helping with injecting insulin and/or purchasing, supporting and reminding the women to take the treatment prescribed. Six of the women reported moving to their own parents’ household during their pregnancy as their parents would ‘be giving them better care’.

#### 3.2.2. Coordination with Work and Social Life

Adhering to the treatment plan was also described by many women as challenging because it required coordination with work, whether it was household work in the rural setting or formal employment in the urban setting, and taking part in social life in the community. As described, the diet was experienced by some women as making them tired, and for some it hampered their ability to perform their work well—whether in the formal sector or in their household. Thus, the women would daily have to balance following the treatment correctly with carrying out their work, including having the stamina to perform it well.

The treatment advice on preparation of alternative foods and splitting the food into several meals throughout the day was highlighted as particularly challenging by all women. Cooking and meal timings had to be carefully coordinated with the women’s household duties, with the schedule of the rest of the family and for some also with their formal jobs. Moreover, preparing the recommended food was considered cumbersome.

‘*Now it is three of us living here, and in the morning, I have to cook and pack food for my son, so then I have to cook only for my sake in the noon and I have to sit and clean those greens. It’s a difficult thing. Daily they have told me to eat greens, but I am taking it only twice a week, because it is very difficult for me to sit on the floor and clean the greens.*’

(Woman no. 8, Chennai)

Consequently, eight of the women admitted to not always adhering to the timings and the splitting of meals recommended by their health care provider. Among the women in Chennai, three of them were currently employed in the formal sector, and for them it could be a challenge to pack and bring their food from home as they could not get the recommended food at work, and also to eat several meals throughout the day rather than the standard lunch break.

‘*The biggest change is taking time out during work to sit and eat. Another big change is to plan what to have and at what time, and then pack everything and take it to work. Probably if I was at home it is a different thing, but because I come to work, I need to figure out: this I’ll have for my 11 o’clock break and this I will have at 3 o’clock. So that is a bit difficult. I have to take time out to think what I need to eat [...] I am not able to follow it [the diet] all the time. I would say probably at least four days a week I am able to follow the diet properly. Otherwise it is not really possible.*’

(Woman no. 10, Chennai)

Social life could also be affected by the treatment. Half of the women said they did not go out as much as they used to both due to their pregnancy, but also because they experienced it as impossible to follow the diet when eating in restaurants or going for social functions.

‘*The main thing is my food, which has been restricted. Before, when we went out, I was able to eat out, but now I can’t eat in the hotels [restaurants] […]. It is like a jail for me, and I don’t go out. This is difficult and I feel upset to be like this.*’

(Woman no. 8, Chennai)

Others did not restrict themselves from participating in social events and gatherings, but then admitted to finding it difficult to resist eating the food that was offered even though it was not ‘allowed’ in their diet. It was also considered socially inappropriate to decline a meal offered by others, which could raise critical questions and unwanted attention.

‘*If I go out—the sympathy people show when they are like ‘you are not eating’. I mean the mind-set our people have is like ‘oh you need to eat more’. […] I try to avoid going to parties; if I go, there are so many things put up. I am okay with avoiding it because I have convinced myself in my mind, but people keep on showing me that sympathy which makes me feel bad. […] There were so many people around and everyone was bringing food for me, like you know ‘have this, have that’, so that was so tempting because those were all fried items and sweets.*’

(Woman no. 6, Chennai)

Women who were taking metformin generally noted that they found it convenient and had no challenges with taking it in terms of coordination with their everyday life. Two of the urban women taking insulin (Woman no. 6 and Woman no. 19) noted that it could be challenging or at least awkward to go aside and inject the insulin when at social gatherings or at the office.

### 3.3. Health System Level Challenges

#### 3.3.1. Experiences with Availability and Cost

Issues of availability and cost of the treatment, both foods and medication, were impediments for women in both Thiruvallur and Chennai, but especially presented a challenge for the women in the rural area. Many of the women noted that the recommended diet was largely a ‘usual South Indian diet’, meaning that there were no foreign or exotic contents in the meal plan, and all the recommended food items were part of their traditional diet. However, the amounts and proportions changed, and the items that were recommended were for some women not only more cumbersome to prepare, but also less available in their local community.

‘*They told me to eat a lot of vegetables and then greens, but I have not eaten it. I didn’t get that much of greens to buy and eat. I have only not eaten it, because I didn’t find any of the greens here.*’

(Woman no. 9, Thiruvallur)

The women were recommended to substitute rice with ‘tiffin’—a light meal consisting of chapatis (wheat-based pancakes), dosas (pancakes made from fermented rice and/or lentils) or idlis (small ‘cake’ made of different types of lentils and rice) with curry sauces, dal (lentil dish) or vegetables and with chutneys (e.g., coconut, tomato, tamarind, mint). Cooking these types of meals presented an extra cost for most women compared to the everyday rice-based meals. Woman no. 7’s family had experienced various adversities in recent years and was therefore in a financially difficult situation during her pregnancy. Talking about her diet, she said the health care provider had told her to eat tiffin, but:

‘*We don’t have that much money so now and then I eat rice. […] If I eat rice, I just have to cook it and then I can eat it, but for the tiffin I have to grind an extra chutney for it. [...] I eat tiffin only twice a week. To make tiffin is so difficult and my husband is a daily wage earner, so it’s difficult for us to buy many things to eat. […] Buying fruits are expensive and then to make tiffin we need wheat flour, which is expensive. We get 20 kilos of rice and dal from the ration shop from the government, so many days we are just eating that.*’

(Woman no. 7, Thiruvallur)

Among the women who were taking insulin, there were diverging opinions on the availability and cost of it depending on the woman’s situation. Insulin should be freely available to women with GDM in the government health system; however, as per the women’s accounts, obtaining it could be associated with difficulties. Woman no. 5 was living in Thiruvallur with her husband, a daily wager, and was experiencing her second pregnancy affected by GDM. She had been instructed to take insulin and described her struggles to get it:

‘*[The health care provider in Thiruvallur] told me that they give free injections and medicines at the General Hospital in Chennai, but I went there 3–4 times and we spent money. We spent around 500 rupees, and we didn’t get the insulin at all. […] When I went there and asked for it they shouted and said ‘who told you that we give you insulin? We don’t just give. If you want, get admitted here.’ [...] My husband used to go two days to work and the next day he used to take leave [to go to the hospital], so we really felt it hard and struggled a lot. Whenever he takes leave, they cut his salary.*’

(Woman no. 5, Thiruvallur)

Getting insulin free of charge required the women to be admitted at the government hospital in Chennai for up to one month until their glucose levels were under control. While the treatment there is free of charge, being admitted at the hospital in Chennai was associated with indirect costs such as loss of income and transportation. Being admitted was further difficult if there were other children and no family to look after them in the meantime. After struggling to get the insulin for free via the government system, Woman no. 5 and her husband finally gave up and decided to buy it themselves through the local pharmacy. This put a financial strain on the family as the cost of the insulin treatment amounted to around three weeks’ salary.

#### 3.3.2. Experiences with Interaction with Health Care Providers

Some women who expressed concerns about the treatment, as described earlier, said that they accepted it after talking to their health care provider, who had reassured them that it would be safe for them and their baby.

‘*It [insulin] is good for my child, so I was okay to take it… She [the doctor] explained me everything: why I am taking this and how it affects the baby and everything. So, I was clear about why this ‘procedure’ is [necessary], and so I was okay with that.*’

(Woman no. 19, Chennai)

The interaction and communication with a health care provider, who provided adequate information and conveyed the message that taking the treatment would not harm the fetus, was thus a facilitator for overcoming the women’s reluctance. In particular, six of the women in rural Thiruvallur gave very positive accounts of their interactions with the health care provider in charge of giving the dietary counselling. The women described how the health care provider would help clarify any question they might have, but also how he respected the emotional reactions of women and consoled them and motivated them with a positive attitude to follow the treatment. Sometimes he would extend his role as the woman’s health care provider to include the woman’s family members and convince them to support the woman to follow the advised treatment.

However, interactions with health care providers were sometimes also experienced as challenging and an impediment for following treatment. Although none of the negative situations were directly related to their treatment for GDM, several of the women from Thiruvallur described instances where health care providers shouted at them, accused them of lying when the women had approached them with a problem, or had exerted pressure on the women in order to get them to accept a treatment or procedure. In Chennai, the two women who only had primary school education felt intimidated by the environment at the hospital; both described being afraid to ask the doctor questions even when they were in doubt about something regarding their treatment for GDM.

‘*If I go inside to do the tests in the government hospitals, I am not supposed to take anyone along with me. I should go alone for the tests. Even to visit the doctor I have to go alone. […] I feel it very difficult to go inside to do the tests and I don’t ask any questions. I just go and do the tests and go away. I will be afraid to go inside and to see the doctors, but my family will tell me to go there and ask for things.*’

(Woman no. 1, Chennai)

Moreover, confusion or doubts sometimes arose as a consequence of the interaction with health care providers. This occurred when women met different health care providers, who gave them conflicting or diverging messages. Woman no. 13 from Thiruvallur was initially diagnosed with GDM in her fifth month, but she was not able to go to the health center to receive the results of the diagnostic test herself. Instead, her husband picked up the result written in a notebook. She then went to her local village health nurse with the results and was told the results were ‘normal’, which gave her the impression that she did not have GDM after all.

‘*I showed the results to the nurse when she came to the village for the visit. She looked at the results and said it was normal… [So] I told myself that I don’t have sugar [diabetes] and I don’t have to take the tablets, so I didn’t go and take the tablets.*’

(Woman no. 13, Thiruvallur)

Two of the women stopped taking their medication and one stopped going to the checkups after advice from different health care providers subsequent to the diagnosis. Woman no. 12, for instance, was taking insulin until her 8th month when she went to another doctor, who told her to stop taking it. Later, when she was admitted to a government tertiary hospital, she was once again instructed to take insulin.

## 4. Discussion

In our study of women’s experiences with the dietary and pharmaceutical treatment for GDM in rural and urban Tamil Nadu, India, we found that the women experienced substantial emotional and social difficulties when trying to manage the treatment at home, in their social environments, and when encountering the health system.

We found that the women generally reacted to the diagnosis with shock, fear and anxiety for the health of their baby, a reaction that has been documented in several other studies from across the world, including in settings as diverse as China [26], Vietnam [27], the United Kingdom [28], Sweden [29], Australia [30,31], Canada [32], Austria [33], Iran [34], the United States [35] and Denmark [36]. The concern for the wellbeing of the baby was a strong motivating factor for treatment adherence, but the women—both in the urban and the rural areas—nonetheless perceived treatment as an ordeal.

An important barrier for initiating treatment, especially pharmaceutical, identified in our study was that at times following the treatment was seen as counterintuitive to what was considered popular belief about what is healthy and safe during pregnancy, such as potentially adverse effects of medications. In their study on the lived experiences of women with GDM in the U.S., Gray et al. also noted that women were concerned about the short- and long-term effects of insulin treatment on infant’s health [37]. As shown in our study, apprehension about insulin’s negative implication on the health of the fetus could result in treatment delay.

In our study, women also expressed concerns about the recommended diet, including fears that it would ‘not be enough to nourish the fetus’ or themselves. Fear that diet control will lead to nutrition deficiency for the fetus has been raised as a challenge in other contexts, including one study on the experiences of South Asian women with GDM in Australia [30], and studies among women in China [26], Iran [34] and Canada [38]. Such beliefs are important to address in a clinical setting and highlights the importance of tailored educational messages to the woman and her family.

Moreover, dietary recommendations require scheduling and preparation. In our study, we noted that adherence to recommendations required women to carefully coordinate their work and social life sometimes in complex family set ups where they hold little opportunity to negotiate nor change their behavior. This has also been reported in other studies. Thus, food timings conflicting with work schedules were documented in studies from Iran [34] and Canada [39], and dietary regimens resulting in social isolation have been shown across a number of different contexts, including Australia [31,40,41], Canada [39], Sweden [29], United Kingdom [28], United States [37] and Iran [34]. In their study of Iranian women’s experiences with GDM, Ghaffari and colleagues, for instance, showed that the dietary restrictions could result in self-social isolation due to stigma associated with the condition—a condition that would be revealed by following the dietary restrictions in the presence of others [34].

The evidence suggests that as women gain mastery of self-management of treatment, whether dietary or pharmaceutical, and it becomes part of daily routine, the perceived hardship associated with treatment diminishes [31,42]. Women in our study described a similar process, highlighting that in the absence of more persistent barriers and with proper social and health care provider support, the hardship may be overcome, and coordination experienced as less problematic.

Another critical issue identified in our study was that of the cost and availability of both recommended foods and insulin for people with limited resources, especially in the rural area. Insulin is on the national list of essential medicines in India, and therefore is claimed to be easily available and accessible at all levels of health care in the country [43]. However, we found that some of the women requiring insulin reported difficulties in obtaining it free of charge from the government system due to associated conditionalities involved. Ghaffari et al., in their study of Iranian women’s compliance with GDM treatment, similarly noted difficulties related to the hospitalization of women with GDM needing insulin, including anxiety, lack of family support and problems with being away from one’s family [34]. In our study, the involved financial and social costs of hospitalization were a burden to the entire family for the women needing insulin from the government system. Thus, the policy that women requiring insulin should be admitted until their glucose levels are normalized may have a valid clinical reasoning but ignores the impediments and social and indirect financial costs for the affected women.

Interestingly, although the recommended dietary regime was generally described as a ‘usual South Indian diet’, its composition made it difficult to follow for women of little means and/or in more remote rural areas. For many families in Tamil Nadu, especially those depending on daily wages and who get free or subsidized rice from the government, rice is the only affordable diet. The adverse impact on the family’s economy is even further aggravated if the women needed insulin as well. Various other studies have highlighted that existing dietary recommendations for women with GDM can be a financial struggle and are not always culturally appropriate for women from non-Western cultures, where these women struggle with appropriate substitutes and variation [27,28,30,34,40,44,45,46]. Accordingly, there continues to be an urgent need for further ensuring that the dietary recommendations for women with GDM are not only culturally compatible but also take into account the local availability and cost.

The role of others, whether it be the women’s health care providers, family or other social relations, was found to be an important aspect influencing the women’s treatment. We saw that positive interactions with health care providers could lessen the women’s concerns over side-effects and facilitate motivation for the treatment. In her study of obese women with GDM in the UK, Jarvie likewise note that a good relationship with the community midwife made women feel comfortable in discussing weight and lifestyle issues [44]. However, in ours, Jarvie’s and other studies, negative interactions with health care providers also seem to be a substantial impediment for the women [29,34,37,44,47]. Experiences of being shouted at, reprimanded, or judged hardly facilitate empowering, trusting relationships and motivation. In addition, some women were confused over the messages conveyed by the health care providers or even received erroneous or conflicting information. Kapur et al. reported that dietary change requires giving up long established patterns of eating behavior and acquiring new habits [48]. ‘Non-compliance’ to diet advice may be a result of the inability of the health care system to provide diet self-management training for women with GDM. Based on this study’s results, we suggest that adherence is enhanced with diet counseling that is supportive, easy to understand and practically possible to follow in women’s daily lives and in the social setting they live in. This would require continued capacity building in the form of enhancing both clinical and communication skills to ensure that health care providers are properly equipped to provide the needed information and motivation to facilitate self-management for women with GDM.

The women’s family, including her in-laws, was a very strong influencing factor for the women’s ability, motivation and opportunity to follow the treatment. Family support is very important in dietary adherence, particularly if food does not have to be cooked separately and the whole family eats the same food [48]. Support from the family could mitigate many of the other barriers, but the family can also enhance them. The importance of social support in GDM management has been highlighted by a number of other studies as well [28,29,31,34,39,40,49]. In our study, the burden of the required coordination was, for instance, eased if the women had practical support with household chores, or if the rest of the family agreed to follow the same diet as her, which also lessened experiences of temptations. The cultural notions of appropriate diet for pregnant women and the financial costs associated furthermore meant that the treatment for some women had to be negotiated in the family. In this context, it is vital for health care providers to consider the cultural perceptions of the women as the main caretakers of the entire family, which creates burdens for the women. It is also important for health care providers to acknowledge the role of daughters-in-law, including their often low decision-making power and possibilities to negotiate, e.g., which foods are eaten, in their families

To the best of our knowledge, our study is the first to make such an exploration among women in India, despite the fact that around three to five million births in India may be affected by GDM annually. While similar studies from low- and middle-income countries, such as China, Vietnam, Thailand, Zimbabwe and Iran [26,27,34,50,51], have been published in recent years, the majority of studies focusing on treatment experiences of women with GDM are from high income countries [13,14]. This highlights a substantial knowledge gap in available evidence. Utz et al. showed that GDM guidelines and management are missing in many low- and middle-income countries and argued for strengthening of health systems to address the burden of GDM [52]. Consequently, efforts continuously need to be scaled up and strengthened to understand and address the individual, health system, societal and structural barriers to treatment that women with GDM are facing, especially in low- and middle-income countries. Our study particularly suggests that social support and competencies to navigate in the health system and process health- and treatment-related information are crucial aspects. In future studies, including intervention studies, it would therefore be relevant to focus on the involvement of family members and/or peers as well as health literacy.

While the study provides important and much needed insights into the barriers and facilitators to treatment as experienced by women with GDM in rural and urban Tamil Nadu, it has some limitations. First, in our study we interviewed only women diagnosed with GDM. It would have strengthened our findings if we had been able to triangulate data sources [53], e.g., interviewed health care providers about their experiences as well. Second, most interviews were carried out with the assistance of an interpreter. The challenges, but also potential advantages, with cross-cultural research are notable [54]. We tried to diminish the limitations associated with interpretation through careful training, discussions of interviews and verbatim transcriptions and translations. Also, the interviews were undertaken by a Danish female researcher (KKN) with assistance from a Tamil female translator, which may have further enabled communication across cultural and social barriers. The coding and interpretation of data beyond the transcriptions and translations were carried out by KKN. When conducted by an ‘outsider’, there may be social and cultural aspects or details that were overlooked in this process due to unfamiliarity with the context. On the other hand, cultural ‘insiders’ may also be biased and ‘too close’ to the culture to ask critical questions or discover new patterns in data. To further limit cross-cultural misunderstandings or misinterpretations in the later stages of the analysis, categories and presentation of findings were discussed and critically revised by the two Indian co-authors (AK, VS).

Finally, an important aspect of undertaking qualitative research and ensuring trustworthiness and reflexivity is to acknowledge and consider the researchers’ preconceptions and positions [25,55]. The study was part of a larger mainly biomedical research project focusing on GDM screening and treatment and the authors all have backgrounds within public health science or medicine. This meant that we had a particular interest in understanding issues that would act as barriers and facilitators for effective treatment. As the production of qualitative knowledge is inherently linked to the researcher(s) preunderstanding and professional backgrounds [55,56], other researchers with a social anthropological background might therefore have paid more attention to other concepts and patterns in their analysis and interpretation.

## 5. Conclusions

Our study investigated women’s experiences with the dietary and pharmaceutical treatment for GDM in rural and urban Tamil Nadu, India and identified a number of aspects influencing both dietary and pharmaceutical treatment of GDM. Some of these mainly acted as barriers; in particular, treatment being considered as a struggle and counterintuitive to lay perceptions about what is considered safe and healthy, hardships associated with the treatment including difficult interactions with health providers and the required coordination with work and social life. However, we also found that social support and positive, high-quality interactions with health care providers could mitigate some of these barriers and facilitate the treatment process. Greater efforts at awareness creation and systemic adjustments in care delivery targeting the individual, family, community and health system are needed in order to ensure that women with GDM both have the opportunity to access treatment and are enabled and motivated to follow it.

## Figures and Tables

**Table 1:** Background information on the participating women diagnosed with GDM.

Participant ID	Location	Age	Educational Level	Employment	Treatment during Pregnancy	Obstetric History at First Interview	Number of Pregnancies with Diagnosed GDM	Timing of Interview(s)	Number of Years Married	Religion
1	Chennai	38	8th Grade	Housewife	Diet, metformin and insulin	First pregnancy. Was originally a twin pregnancy, but one fetus got aborted	1	7th and 8th month of pregnancy, and 2.5 months after delivery	1 year	Christian
2	Chennai	28	Graduate	Employed but on leave	Diet, metformin and insulin	Two abortions	1	6th month of pregnancy and 1 month after delivery	1 year	Christian
3	Thiruvallur	24	10th Grade	Housewife	Diet	First pregnancy	1	9th month of pregnancy, and 4 months after delivery	1 year	Muslim
4	Chennai	26	Graduate	Housewife	Diet, metformin and insulin	One stillbirth	2	9th month of pregnancy	1.5 years	Christian
5	Thiruvallur	24	10th Grade	Housewife	Diet and insulin	A 1-year-old daughter	2	3rd and 8th month of pregnancy, and 5 months after delivery	2 years	Hindu
6	Chennai	26	Post-graduate	Employed	Diet and insulin	First pregnancy	1	6th month of pregnancy	2.5 years	Hindu
7	Thiruvallur	31	9th Grade	Housewife	Diet	An 11-year-old daughter and had difficulties in conceiving a second child	1	9th month of pregnancy and 4 months after delivery	13 years	Hindu
8	Chennai	32	10th Grade	Housewife	Diet and insulin	A 9-year-old son, and had difficulties conceiving a second child	1	8th month of pregnancy and 3.5 months after delivery	11 years	Hindu
9	Thiruvallur	24	10th Grade	Housewife	Diet	A 1.5-year-old daughter and one abortion	1	7th month of pregnancy and 2 months after delivery	2.5 years	Hindu
10	Chennai	29	Post-graduate	Employed	Diet and metformin	First pregnancy	1	8th month of pregnancy	1 year	Hindu
11	Thiruvallur	28	10th Grade	Housewife	Diet	Two daughters 3 and 1.5-years-old. Had difficulties conceiving the first five years of marriage	2 (3)	1.5 years after delivery following last reported GDM diagnosis, but now 8th month pregnant	9 years	Hindu
12	Thiruvallur	25	12th Grade	Housewife	Diet and insulin	Twins who died shortly after the delivery, one 1.5-year-old son and now pregnant for the third time	1	1.5 years after delivery following last GDM diagnosis, but now 7 months pregnant without GDM	4 years	Hindu
13	Thiruvallur	28	10th Grade	Housewife	Does not believe she had GDM	A 5-month-old daughter	1	5 months after delivery	2 years	Hindu
14	Chennai	30	Post-graduate	Housewife	Diet and insulin	A 6-year-old son, one abortion, and a 2-months-old daughter	1	2 months after delivery	6 years	Hindu
15	Thiruvallur	30	10th Grade	Housewife	Diet	First pregnancy	1	8th month of pregnancy	9 months	Hindu
16	Chennai	33	Post-graduate	Housewife	Diet and metformin	Two abortions, a 9-year-old son, a 7-year-old son and a 3-months-old son	1	3 months after delivery	10 years	Hindu
17	Thiruvallur	27	5th Grade	Housewife	Diet, metformin and insulin	Has a 3-year-old daughter and a 1-month-old daughter	1	1 month after delivery	4 years	Muslim
18	Thiruvallur	27	9th Grade	Housewife	Diet	First pregnancy	1	9th month of pregnancy	9 months	Hindu
19	Chennai	30	Post-graduate	Employed	Diet and insulin	Has a 4.5-year-old daughter and a 1-week old daughter	2	1 week after delivery	5 years	Christian
